# Persistent Hallucinations in a Middle-Aged Man After COVID-19 Infection

**DOI:** 10.7759/cureus.73570

**Published:** 2024-11-13

**Authors:** Bárbara L Mesquita, Daniela Jeremias, Ana Margarida Fraga, Joana Romao, Sofia Paulino

**Affiliations:** 1 Department of Psychiatry, Hospital Santa Maria, Lisbon, PRT; 2 Department of Psychiatry, Hospital de Egas Moniz, Lisbon, PRT; 3 Department of Psychiatry, Hospital de Cascais, Lisbon, PRT

**Keywords:** auditory hallucinations, covid-19, covid-19 neuropsychiatric manifestations, near-death experience (nde), suicidal ideations, suicide and depression

## Abstract

The COVID-19 pandemic continues to surprise us all with its aftermath. The connection between the virus and neuropsychiatric symptoms is thought to arise from both direct and indirect impacts on the brain, and various explanations have been proposed to account for these effects. This is a case of visual and persisting auditory hallucinations in a middle-aged man, with no previous psychiatric illnesses, after suffering from very severe symptoms of COVID-19. These hallucinations remained for months following recovery from the virus. While hospitalized, the patient also reported having a near-death experience and seeing his deceased father. Despite the inevitable time frame limitation when it comes to severe neuropsychiatric manifestations of patients who were diagnosed with COVID-19, it is imperative to research adequate and effective interventions.

## Introduction

COVID-19 is an infectious disease caused by a virus that was first detected in Wuhan, China, in December 2019. Coronavirus was declared a pandemic by the World Health Organization (WHO) on March 11, 2020 [1]. It can cause a wide range of symptoms, from none at all to mild respiratory symptoms and to fatal severe acute respiratory syndrome (SARS) [2].

The global spread of the virus has had a considerable negative impact on the mental well-being of people all around the world. A significant amount of the psychological manifestations of COVID-19 are a result of fear of the illness itself, public restrictions, and a mass quarantine, which has led to a hazardous social isolation period. Nevertheless, the viral illness itself may have neuropsychiatric manifestations, including psychosis [3,4].

Psychosis is a state in which the individual experiences a severe disconnection from reality or a loss of ego boundaries. It is primarily expressed by the presence of hallucinations or delusions. Therefore, it can be considered to be a set of symptoms in which a person’s mental capacity, affective response, and capacity to recognize reality and relate to others are impaired [1]. A hallucination is a perception without an object. They are not distortions of real perceptions and can occur simultaneously alongside real perceptions. Hallucinations can be auditory, visual, olfactory, gustatory, and bodily sensations. A pseudo-hallucination is similar; however, the difference from hallucinations is that the patient can have insight and/or have it in the inner space, which means reporting these inside their head (unlike hallucinations that occur in the outer space).

A growing number of evidence suggests that the virus can precipitate psychosis among infected individuals [3]. However, the etiology of COVID-related psychosis remains unclear, and much more research is needed [5]. Common themes observed in these patients include severe anxiety, agitation, suspiciousness, and auditory hallucinations [3].

In recent literature, there have been several proposed mechanisms to try and explain this association. One of them is that both SARS-CoV-1 and SARS-CoV-2 can use the cell membrane-bound angiotensin-converting enzyme 2 (ACE2) for cellular entry, which, among other tissues, is expressed on vascular endothelial cells in the brain. On the other hand, according to recent studies, SARS-CoV-2 seems to have an even higher affinity to bind to this receptor than SARS-CoV-1 [6]. Therefore, SARS-CoV-2 can migrate to the brain by disrupting the blood-brain barrier (BBB), interact with the ACE2 receptor, expressed by brain tissue, and therefore be a cause of various psychiatric and neurological symptoms [7-10].

Another proposed mechanism, known as “cytokine storm,” implies the systemic inflammatory response associated with severe cases of COVID-19 pneumonia. According to this theory, the peripheral activation of proinflammatory cytokines (e.g., interleukin (IL)-6, tumor necrosis factor (TNF)-alpha, IL-8, IL-10, IL-2R) in response to COVID-19 infection may contribute to neuroinflammation via increased permeability and compromised integrity of BBB, which enables the virus to enter the CNS, resulting in neurological manifestations associated with COVID-19 [3,4,8]. Increased levels of IL-6 have been detected in cerebrospinal fluid patients with schizophrenia, and its high levels in young individuals have also been related to the development of psychosis [10].

Another possible mechanism of psychosis in COVID-19 patients may be related to severe sensory deprivation, which can occur because of subsequent isolation protocols in a single hospital room with minimal direct human contact. Similar cases of acute psychosis have been described in prisoners in solitary confinement and in experimental sensory deprivation [3].

Additionally, according to some studies, psychological stress can trigger psychosis in vulnerable individuals through increasing the levels of dopamine levels in the brain, and therefore causing psychosis. It is reported that subjects with high anxiety show more uncertainty when processing their sensorial experience, hence being more prone to experiencing visual and auditory hallucinations when under extreme anxiety. In addition, major traumatic life events also increase the risk of psychotic disorders [11].

Iatrogenic factors should also be taken into consideration. Psychotic symptoms are documented adverse events associated with corticosteroid use [12]. In fact, during the SARS-CoV-1 pandemic, multiple patients who were treated with high doses of steroids consequently experienced hallucinations and symptoms of mania, which subsided after treatment was stopped [13]. However, the definitive pathogenic mechanisms involving CNS invasion remain undetermined [2,10,13].

We present the case of a patient with no premorbid psychiatric history who developed auditory and visual hallucinatory activity associated with interpretations of a delusional nature during his stay in an intensive care unit. This clinical presentation did not subside after full recovery of his consciousness nor following several months of psychiatric evaluation and intervention.

## Case presentation

The patient is a 51-year-old married man. He had a previous medical history of hypertension, diabetes type II, dyslipidemia, hyperuricemia, and obesity. No history of personal or family psychiatric or neurologic disorders was reported.

At the beginning of August 2020, the patient was diagnosed with COVID-19, and on the 16th of the same month, due to the rapid deterioration of his symptoms, with high fever, difficulty breathing, and muscle pain, he was admitted to the emergency department of the Cascais Hospital. He was hospitalized with the diagnosis of SARS-CoV-2 pneumonia and began an empirical antibiotic treatment with amoxicillin-clavulanic acid and dexamethasone. Days after his hospitalization, the patient developed acute respiratory distress syndrome (ARDS), and he was transferred to the intensive care unit, where he was intubated and mechanically ventilated. Because of his continuously rapid worsening of symptoms, he was transferred to the São José Hospital and underwent extracorporeal membrane oxygenation (ECMO) from the 23rd of August until the 25th. He developed delirium and ventilator-associated pneumonia. On the 2nd of September, he was transferred to the São Francisco de Xavier Hospital to receive dialysis, which occurred without complications. On the 9th of September, he was readmitted to the Cascais Hospital and was discharged soon after.

On the 17th of December, the patient attended a psychiatry consultation to address symptoms of depressive humor, anhedonia, as well as physical and psychological anxiety. He denied suicidal ideation at this point but reported nihilistic thoughts. He claimed to be able to see and talk to his dead father. When describing it in more detail, the patient reported that these symptoms started during his stay at São José Hospital by stating, “When I woke up in the hospital bed, it was weird, and I don’t know if I lived it or if it was all a dream, but I got up and ran through this countryside scenery, with lots of beautiful trees around me and the grass of the most beautiful green I had ever seen. I ran and ran until I got to a huge gate that opened up for me, and as I was going in, I looked down and saw myself lying on the grass, dead, with parts of my body burned. At this point, I remember feeling quite disappointed with my son for not making sure I had been cremated properly. As I continued walking through the field, I saw my dad, who was a shepherd. I tried talking to him, but he kept on saying, ‘Get out of here, son, it’s not your time yet.’ And I felt sad that he didn’t want me to be with him, so I asked, ‘Why? Don’t you love me anymore, Dad?’ I really enjoyed seeing my dad; I felt like it was real.” He references seeing his dad recurrently while he was at the hospital, even saying he would always ask for a blanket for his dad, who “was always cold” and slept with the patient on his bed right next to him.

He continued saying, “I had another dream where I was in Nazaré, and my dad grabbed me and asked what I was doing there.” He also claimed to have maintained visual hallucinations of his dad since discharge. “I sometimes, after going home, would go for long walks with my dog in the woods behind my house and would have long talks with him about everything… even soccer!” He went on by saying that he feels his hand on his shoulder almost every day.

Despite the patient describing alterations of the sensorium-perceptive sphere in an apparent oneiric context and associated with an alteration of the state of consciousness, the perceptions were not only extremely vivid but also described in detail and associated with an absence of insight; therefore, it seems unlikely that this is the result of an illness. These symptoms persisted even after the complete recovery of the patient’s state of consciousness and reintegration into his socio-family environment. 

The patient also reported to have been hearing voices inside his head since being committed, “during my stay at the hospital, I saw myself on the other side and spoke to dead people. Since then, they are here with me, waiting for me to cross over. I haven’t stopped hearing their voices ever since. It seems like there are people talking in my head at all times. I hear them say that my time has come, but when I ask what they mean by that, they never answer. They are always talking to each other about me, but when I go near them, they stop talking.” Because of the patient’s visual hallucinations, he was proposed for a neurological consultation, which ended up being differed multiple times because of the pandemic. After psychiatry evaluation, he started fluoxetine 20 mg, alprazolam 0.25 mg, trazodone 100 mg, and risperidone 1 mg. 

On the 5th of January 2021, he was admitted to the emergency department following a suicide attempt. The patient was found with a rope by his son and wife after being alerted by their dog. The mental status examination revealed a depressed mood. The patient also described maintaining complex/scenic visual pseudo-hallucination and suicidal thoughts with a plan already mapped out. It was proposed for the patient to be committed, but he refused. However, he accepted to go to a psychiatric consultation. He was then discharged from the hospital against medical advice with the same psychiatry medication plus olanzapine 5 mg, as needed.

After 10 days, upon psychiatric revaluation, the patient maintained extreme sadness (“I have no joy in living anymore.”), visual hallucinations with his dead father, hearing voices in his head (auditory hallucinations), and suicidal ideation. Because of a COVID-19 outbreak in the psychiatric wing, he was once again not committed to the hospital. It was agreed that his wife would watch over him at all times and that a re-evaluation would be scheduled for the earliest possible date. Fluoxetine was augmented to 40 mg, olanzapine to 10 mg, and risperidone 1 mg was maintained. 

On the 4th of February, the patient was once again re-evaluated. He reported not being able to see his father anymore nor sustaining a conversation with him, claiming that he only tells the patient “not to go inside the portal of death.” However, he was still able to feel his father’s hand on his shoulder on occasion. He maintained auditory hallucinations, stating, “There are still people talking inside my head; I can’t take it anymore. They keep saying I should kill myself, and they never shut up. I am really not doing well.” He also maintained severe depressive symptoms, “I feel like I am someone else; I can’t find joy in anything anymore.” and suicidal thoughts, “I wish I could go join my father, but my son keeps telling me that he needs me. I have thoughts about dying all the time.” In addition, he described panic-like episodes, with feelings of a “squeezing” in his chest, tachycardia, blurry vision, tremors, and sweating. After the re-evaluation, the prescribed fluoxetine was augmented to 60 mg/day.

Over the following months, despite multiple therapeutic adjustments and a discrete mood and anxiety improvement, the hallucinatory activity persisted on a daily basis without asymptomatic periods. The hallucinations became progressively more insufferable and incompatible with socio-occupational and family functioning for the patient. There was always a prevalence of a clinical alteration of the sensorium-perceptive sphere that was polymorphic. In this case, the patient was alternating between hallucinatory activity that was essentially visual at the beginning, posteriorly tactile, and ultimately auditory, meeting criteria of acoustic-verbal hallucinatory activity, and other times of auditory pseudo-hallucinations.

## Discussion

This case demonstrates how acute psychosis in a patient with no previous psychiatric history can emerge from a severe COVID-19 illness. The patient’s auditory and visual hallucinations started during his stay at the hospital, and while the visual hallucination of his dead father eventually subsided with antipsychotic medication, his auditory hallucinations still remain to this day. The exclusion of a neurologic cause would have been of extreme importance, given that an organic cause should be excluded in the presence of visual hallucinations (which indicate an organic diagnostic, unlike auditory hallucinations that include a psychiatric diagnosis) and that the hallucinations did not subside with adequate treatment. However, neurologic evaluation and new head imaging were repeatedly postponed because of frequent cancellations from the specialty due to constant changes in hospital measures caused by a third wave of COVID-19 infections. In this context, a new head CT was requested, and it was proposed commitment in the psychiatric ward, which the patient refused. The patient additionally started developing symptoms of post-traumatic stress disorder associated with his COVID-19 infection commitment. Finally, on the 25th of March, a head CT was made, which came back with no significant changes.

There have been several studies on the development of neuropsychiatric symptoms in patients who tested positive for COVID-19. Similarly, the other coronavirus epidemics, namely, SARS-CoV-1 and Middle East respiratory syndrome coronavirus (MERS-CoV), have been associated with neurological manifestations [13,14]. There is a study by Ritchie et al. (2020) that identified up to 70% of people with confirmed MERS had psychiatric symptoms, including psychosis and hallucinations, during their admission. Additionally, in a study of 90 people with SARS, 59% were diagnosed with a psychiatric disorder, which persisted in a third of the cases two to three years after the illness [15]. In fact, this clinical case’s presentation during the patient’s hospital stay is compatible with the diagnosis of delirium since the patient presented visual hallucinations (as opposed to auditory hallucinations that are more common in a psychiatry diagnosis) and shifts in awareness. However, the psychiatric symptoms persisted as time went by without any changes, resistant to several therapeutic interventions, and now totally independent of any alteration of the state of consciousness.

Previous neurological symptoms identified include headache, dizziness, anosmia, hypogeusia, delirium, viral encephalitis, seizure, stroke, toxic encephalopathy, and acute hemorrhagic necrotizing encephalopathy. Additionally, some psychiatric symptoms described include psychosis, insomnia, aggressive outbursts, anxiety, depression, mania, and trauma-related disorders. The occurrence of these symptoms has been observed more frequently in more severe cases of COVID-19 [7,13].

Psychosis related to SARS and other coronaviruses has been attributed to patients at a higher risk for psychosis from family history, personal history of psychiatric issues, and stressors such as family members being infected with the virus. Given the known similarities in structure between SARS and COVID-19, these similar manifestations were expected [13]. The connection between COVID-19 and new onset psychosis was first observed in China, where a retrospective chart review in the city of Xuzhou showed a 10% increase in psychosis among first behavior abnormalities with the persistence of viral RNA in the brain for several months [3]. In this context, the case study presented is original and relatively uncommon (or underdiagnosed/underreported) since the patient did not present risk factors in his personal or family history, nor circumstances that could unbalance the diathesis between vulnerability and stress.

Apart from the theory that SARS-CoV-2 has the capacity to bind with ACE2 expressed in neurons and glial cells in the central nervous system [7-10], the proposed mechanism known as “cytokine storm” could explain psychotic symptoms since cytokines have also been related to the pathogenesis of primary psychotic disorders [4,9,12]. Immune-based triggers have long been implicated in the pathogenesis of psychiatric illness, including depression, psychotic disorders, and neuropsychiatric manifestations of viral infections [2,3]. In fact, a significant increase in pro-inflammatory cytokines and their receptors has been described in both chronic schizophrenia and the first episode of psychosis. Therefore, we could hypothesize in this particular case that a significant augmentation of inflammatory cytokines from severe SARS-CoV-2 infection could have predisposed the development of the patient’s psychotic symptoms.

In relation to the sensory deprivation mechanism, this patient went through approximately one month of severe isolation with human interactions reduced to the minimum, which could pose as a possible explanation for this patient’s psychosis. However, given that the hallucinations persisted long after leaving the hospital, this might not be the most probable explanation. The same could be said about his corticosteroid treatment, namely, dexamethasone, that even though this could be a cause of the patient’s hallucinations, it does not seem very likely since they should have subsided after stopping the medicine’s use. 

Another probable cause for this patient’s psychosis is the high levels of stress and anxiety he was put through during the time he was committed. His experience at the hospital proved to be very traumatic, as the patient later in consultations showed the development of panic-like symptoms and talked about his stay there with great anxiety and turmoil. Although this could have contributed to the development of this clinical presentation, it does not seem to us that this cause has enough symptomatic relevance that the patient would present so long after being discharged from the hospital. Additionally, the symptoms were resistant to antidepressive, anxiolytic, and antipsychotic therapeutic.

It is also important to discuss matters that the patient’s experience during his hospital stay, where he saw his father and own lifeless body in a countryside scenery, seems to be more in the realm of a near-death experience (NDE) than an actual hallucination like the ones he maintained of his father. According to the literature, an NDE can be defined as the reported memory of a range of impressions during a special state of consciousness. It can include an out-of-body experience (OBE), which constitutes an experience in which a person sees the world from outside of the physical body and may include moving through a tunnel, communicating with light, observing a celestial landscape, meeting with deceased persons, and/or a life review [16]. NDEs have been reported in a multitude of situations, such as cardiac arrest and traumatic brain injury, and also in serious diseases that are not immediately life-threatening, like isolation, depression, meditation, or even without any obvious reason. The NDE is usually transformational, causing enhanced intuitive sensibility, profound changes in life insight, and the loss of the fear of death [17]. In this particular case, the patient was able to see his burned-dead body and talk to his father in a beautiful country scenery while he was going through a severe disease and a tough isolation period.

Although there are neuropsychological, neurochemical, and neurohumoral hypotheses about NDE, it is reported to be a non-pathological experience that involves the psychological processes of dissociation and/or depersonalization as a response to trauma. In fact, several studies claim that these end-of-life dreams and visions (ELDVs) differ in several aspects, from hallucinations to confusional states, which may be associated with the dying process or with medications administered during this time. In a 2010 analysis of deathbed phenomena, it was found that ELDVs tend to be spiritually transformative, while hallucinations tend to be relatively insignificant [18]. It is also important to highlight an integrative study that came to the conclusion that NDE memories are different from imagined autobiographical memories and very similar to memories of real events in terms of detail richness and emotional information [19].

## Conclusions

The time limitation and the critical circumstances in which the COVID-19 pandemic occurred demanded an obvious directing of all efforts to the treatment, prevention, and cure of the infection itself. Now that some time has passed since its most acute phase, it seems quite indispensable to also direct the focus to the neuropsychiatric repercussions of COVID-19, which have shown to be lasting and with a multitude of possible causes for its development.

The neuropsychiatric repercussions of this virus cause immense suffering and completely overshadow a person’s life. They are also apparently maintained in time and can be indefinitely incapacitating despite surviving the infectious disease itself.
